# Barriers and facilitators to physical activity among urban residents with diabetes in Nepal

**DOI:** 10.1371/journal.pone.0199329

**Published:** 2018-06-28

**Authors:** Shanti Kadariya, Arja R. Aro

**Affiliations:** Unit for Health Promotion Research, University of Southern Denmark, Esbjerg, Denmark; University of Birmingham, UNITED KINGDOM

## Abstract

**Introduction:**

Physical activity is an important component of type 2 diabetes management. Physical activity level among general population in Nepal is reported to vary considerably. However, knowledge on physical activity in Nepali diabetics is very limited. Engagement in physical activity could be influenced by perception of barriers against adopting the behavior and benefits of adopting it. This study explores the prevalence of physical activity and factors that promote and hinder the behavior among urban residing diabetic patients from Nepal.

**Methods:**

A descriptive cross-sectional design was adopted using a simple random sampling of type 2 diabetic patients from two diabetes clinics at Lalitpur and Kaski districts of Nepal. Two hundred and seventy participants were surveyed to obtain information on physical activity using Global Physical Activity Questionnaire. Metabolic equivalent values were calculated and categorized into high, moderate and low levels of physical activity. The information on perceived facilitators and barriers was collected by Exercise Barriers and Benefits Survey scale. Odds ratios and 95% confidence intervals of the measures were estimated using multinomial logistic regression.

**Results:**

The study showed relatively high prevalence of physical activity among the urban Nepali diabetic patients; 52% were moderately active and 28% highly active. Travel and work-related activities were the major contributors. Male participants, educated and those living in extended families were more motivated for physical activity than their counterparts. Physical fitness, strength and flexibility, better sleep at night, social interaction and longevity, were identified as the major facilitators. Family responsibilities, busy schedule and family discouragement were identified as barriers against being physically active.

**Conclusion:**

The diabetic patients were mostly moderately physically active. Future research could explore different context-specific ways of remaining physically active, apart from walking and doing household chores. More focus should also be placed on leisure time physical activity as it was found to be low. Interventions could be designed by promoting the facilitators and addressing the barriers of physical activity, which is likely to reduce the healthcare costs of management of diabetic complications.

## Introduction

Diabetes has emerged as one of the most serious global public health challenges. According to the International Diabetes Federation (IDF) estimates, 424.9 million people were living with diabetes in the year 2017 worldwide with a prevalence of 8.8.% among adults between the ages of 20 and 72 years [1]. It is predicted that 628.6 million people will be living with diabetes by the year 2045 [1]. At that proportion, diabetes will already have become the 7^th^ leading cause of death globally by2030 [2].

Nepal is not an exception to the ever-increasing prevalence of non-communicable diseases (NCD). Cardiovascular diseases, chronic respiratory diseases, cancer and diabetes are the four major NCDs in Nepal which accounted for 60% of all deaths in the year 2014 [3]. Likewise, IDF records show that there were 657.200 diabetic patients in Nepal in 2017 [4] with an estimated prevalence of 14.6% among urban residents aged 20 years and 2.5% among those living in the rural areas of the country [5].

Diabetes is a chronic disease characterised by elevated level of blood glucose. Type 2 diabetes is the most common type of diabetes around the world and is attributed to the result of excess body fat, physical inactivity and improper diet [2]. Several studies have already established that physical activity is protective against developing diabetes and reducing the risk of coronary heart disease, stroke, hypertension, cancers (colon, breast) and depression [6–8]. Physical activity has been recommended as a central component of self- management in patients with type 2 diabetes mellitus which helps delay macrovascular complications and premature mortality [9]. Physical activity refers to any bodily movement produced by skeletal muscles that needs energy as input [10]. It includes all daily activities like playing, carrying out household chores, travelling, and activities during work and recreational pursuits. Globally, poor physical activity is the fourth leading risk factor of mortality (6% of deaths worldwide) according to WHO [11].

Even though the effect is known to be more pronounced with structured physical exercise, including aerobic training, resistance training or a combination of both, physical activity when pursued in tandem with appropriate dietary practice is also understood to contribute to declines in HbA1c [12, 13]. Lower HbA1c measures delay the onset and reduction of coronary heart disease, peripheral vascular disease and other clinical endpoints such as nephropathy, retinopathy and neuropathy, which are the commonest complications of diabetes [6]. American Diabetes Association's physical activity protocol for adult diabetic patients states that each diabetic person should do at least 150 minutes of moderate-intensity aerobic physical activity and at least 60 minutes of vigorous-intensity physical activity per week with no more than two days in a row without exercising [14].

The nationwide STEPS survey conducted among general Nepali population in 2013 showed that the majority, i.e. 85% of the respondents were engaged in high level physical activity, around 11.6% were engaged in moderate level activity, whereas only 3.5% were found to be engaged in low level of physical activity [15]. Contrarily, a review of large-scale population level data from 14 Asia-Pacific Countries noted that the prevalence of sufficient physical activity among the Nepalese over 18 years ranged from a low 18% to a very high 92%. This was the highest variation noted among 14 countries that were studied. It is also worth noting that the low 18% prevalence of sufficient physical activity was obtained using GPAQ and the highest 92% was obtained using IPAQ [16]. This suggests a lack of adequate and consistent data that can indicate towards a pattern of physical activity among the general Nepalese population.

Unsurprisingly, there is also a dearth of specific information on the status of physical activity among diabetic patients in Nepal. A hospital-based study was conducted among type 2 diabetic patients in the Midwestern city of Nepalgunj. The study used GPAQ to quantify the measures of physical activity and it found that slightly more than 4 out of 10 (42.1%) of the diabetic individuals were non-adherent to physical activity [17]. Another study among type 2 diabetic patients in central Nepal found that 46% of respondents were non-compliant to exercise advice [18].

Engagement in preventive health behavior like physical activity could, among other reasons, be influenced by the perception of benefits of adopting the behavior and barriers against adopting it. The study from the central region city of Lalitpur found that low self-efficacy and low social acceptability created barriers to being exercise-compliant among diabetic patients. Likewise, laziness, perception that diabetes is not a serious health problem, lack of stamina, and presence of other personal health issues were also identified as barriers to being physically active. Being supported by children and spouse, and being opposed by friends and relatives were noted to have had positive and negative effects on the participant’s physical activity compliance respectively [18].

Additionally, the Nepalgunj based study found that adherence to physical activity was high among Nepalese diabetic patients who belonged to upper middle class and who lived in extended families. Likewise, spouse and family history of diabetes were also found to be major facilitators. On the contrary, divorcee respondents, those living in nuclear family and those who belonged to low socioeconomic class were less adherent to physical activity [17]. Several other personal and situational factors as well as demographic (age, gender, place of residence) and, socio-psychological factors, could influence the adoption of physical activity as health behaviour [19, 20]. Apart from the aforementioned factors, time since diagnosis has also been identified as a factor that affects engagement in physical activity. Likewise, diabetic patients were also found to increase their acts of physical activity after consultation with doctors [21].

However, as stated earlier, there is very limited information available both in the urban and rural Nepali contexts on a) prevalence of physical activity among diabetic patients, and b) the factors known to promote or hinder physical activity among this group of Nepali population. On the pretext of this limited scientific evidence, the study was conducted with the following two aims:

1. To assess the prevalence of physical activity among diabetic urban residents of Nepal
2. To identify the factors that promote or hinder physical activity among diabetic urban residents of Nepal.

## Methods

The methods section has been arranged based on STROBE guidelines.

### Study design and setting

A descriptive cross-sectional study was carried out between February 2016 and November 2016 in Lalitpur and Kaski districts of Nepal. These two metropolises were selected purposively to represent the urban areas of Nepal. Lalitpur is a part of three cities (Kathmandu, Bhaktapur and Lalitpur) within Kathmandu valley that together make up the capital region of the country and Pokhara is the largest city in terms of area, which lies 200 km west of the national capital.

### Participants

#### Eligibility criteria

The inclusion criteria were: male or female diabetic patients within the ages of 30 and 70 years diagnosed with type 2 diabetes at least 3 months before the date of data collection. Patient’s diabetic status was confirmed by their outpatient department (OPD) card.

The respondents in Lalitpur were selected from the privately-run diabetes, thyroid and endocrinology care centre situated in Kupondole and the respondents in Pokhara were selected from the Pokhara branch of the same clinic. These two institutions were selected because of their specific service focus on diabetic patients. The diabetic patients visiting these institutions for their checkup at the days of data collection were individually approached without any previous list at hand. Out of these randomly encountered respondents, those who met the inclusion criteria were asked if they would be willing to participate in the study. The process continued until the desired sample size was achieved. Out of those who were approached and who met the inclusion criteria, agreed to participate in the study.

### Variables

The variables included in the analysis were as follows:

Outcome variables: Physical activity measured by GPAQ
Independent variables: Facilitators and barriers of physical activity measured by EBBS

In the final analyses, the following variables were adjusted for: Age, sex, education, place of residence, income, religion, caste, marital status, occupation and type of family.

### Data sources

Interviewer-administered questionnaire technique was used to interview the participants on their demographic characteristics, socioeconomic status, physical activity and the perceived barriers and facilitators to physical activity. The data collection was undertaken between morning and afternoon, since diabetic patients visited the clinic for getting their fasting and postprandial (PP) blood glucose tested. The data collection was conducted by primary author.

The information on physical activity levels was collected by using Global Physical Activity Questionnaire (GPAQ) [22]. This WHO-developed tool, which has been validated and used in Nepal in the past, is used to obtain information on physical activity participation in three settings (also called domains) as well as sedentary behavior. The tool comprises 16 questions. The three domains that it covers are physical activity at work; travel to and from places and recreational activities. Within the work and recreational domains, questions are sub-divided into two categories defined by the energy requirement or intensity- i.e. vigorous or moderate intensity. To assess the physical activity intensity of an individual, and later to divide them into 3 different categories, metabolic equivalent (MET) score was calculated for each domain.

MET is the ratio of a person’s working metabolic rate relative to the resting metabolic rate. One MET is defined as the energy cost of sitting quietly and is equivalent to a caloric consumption of 1kcal/kg/hour [22]. To calculate MET score, the total minutes spent on physical activity during a day was multiplied with the number of days spent during a week. The value of 3000 MET-minutes per week or more was considered high level physical activity, between 600 and 3000 MET-minutes per week was considered moderate and any value below this was considered low level physical activity.

The information on perceived facilitators and barriers was collected by EBBS (Exercise Barriers and Benefits Survey) instrument along with some additional semi-structured questions which fit the study context [23]. The EBBS questionnaire consists of 43 questions with 29 benefit items. Both benefit and barrier items have been categorized into sub-groups in the tool. Each item in the instrument has four responses that are in forced choice Likert-type format with responses ranging from 4 (strongly agree) to 1 (strongly disagree). The questionnaire was translated into Nepali language prior to data collection.

GPAQ has been used by previous surveys in Nepal to measure physical activity and is well-accepted. The Nepal Health Research Council (NHRC) has also embraced this tool in conducting the STEPS survey in Nepal for studying the risk factors of non-communicable diseases and other associated lifestyle-related behaviours [15, 19]. Although EBBS had not been previously validated in the Nepali context, the tool in the Nepali translated version in this study was found to be understandable and relatable (face validity) to the respondents in a small pilot study conducted by the primary author. So, the scale was chosen for the final study after some minor semantic changes. Cronbach’s alpha coefficient for the benefit subscale was 0.93 and barrier subscale was 0.75 for this study.

The tool was pretested among 10 patients meeting the inclusion criteria at the Diabetes and Endocrinology Centre in Kupandole, Nepal. The results from the pretest were not included in this paper.

### Study size

A total of 270 respondents diagnosed with type 2 diabetes mellitus were included in the study out of all patients who met the inclusion criteria and visited these two institutions between February 2016 and November 2016.

The formula n = z^2^pq/L^2^ was used to calculate the sample size for the study [24] where z is 1.96 for 95% confidence level, L = 5% allowable error and p = 21.3% (q = 100-p) i.e prevalence of adequate physical activity among comparable groups of diabetes patients derived from a similar study conducted in Nepalgunj (p = 21.3%) [17].

Therefore, the sample size of 258 was calculated for this study. To account for potential non-response or incomplete responses, 12 more respondents were interviewed taking the total sample size to 270. Total sample of 270 was divided in such a way that each institution contributed equal number (135 respondents in each) of participants.

### Statistical methods

Statistical analyses were performed using STATA 13 (StataCorp. 2013, College Station, TX). Estimates were expressed as mean, percentage and standard deviation. The respondents were categorized into those with high, moderate and low levels of physical activity based on MET-minutes per day according to WHO GPAQ guideline [22]. Multinomial logistic regression was used for multivariate analyses.

### Ethical approval

Ethical approval for this study was received from Nepal Health Research Council and the Institutional Review Boards of the respective institutions (Diabetes, Thyroid and Endocrinology Care Centre, Kupondole and Pokhara branch). Verbal consent as a means of approval from research participants was clearly mentioned in the ethical approval application. A consent form was included in the beginning of the questionnaire and it was read out to each research participant before every interview. Each research participant was also clearly informed about the purpose of the study. Interviews were made only if they agreed to participate.

## Results

### Socio-demographic characteristics

Table 1 shows socio-demographic characteristics of the study population. A total of 270 diabetic patients were enrolled as the study population with a mean age of 53, ranging from 30 to 70 years. More males were interviewed than females, although not intentionally (62%). Majority (91%) of the respondents were married. Major three ethnic groups, Janajati (40.37%) (Newar, Rai, Magar and Gurung), Brahmin (35.5%) and Chhetri (16.67%) were represented in the sample. Slightly more than 62% of respondents had more than secondary education whereas 12.2% were illiterate. Almost half of the respondents, i.e. 56% belonged to single family. Business was the most common occupation among the respondents (32%), followed by agriculture (17.7%), government/private job (11.48%) and pension (11.48%). The average number of years that the respondents were living with diabetes was 7.5, ranging between 3 months and 34 years.

**Table 1:** Socio-demographic characteristics of urban Nepalese diabetic patients (n = 270).

Characteristics	Frequency	Percentage
**Place of residence**		
Pokhara	140	51.85
Lalitpur	130	48.15
**Gender**		
Male	167	61.85
Female	103	38.15
**Current marital status**		
Married	247	91.48
Single	18	6.67
Divorced	3	1.11
Unmarried	2	0.74
**Education**		
Illiterate	33	12.22
Primary	68	25.19
Secondary and above	169	62.59
**Ethnicity**		
Janajati	109	40.37
Brahmin	96	35.56
Chhetri	45	16.67
Dalit	12	4.44
Others	8	2.96
**Religion**		
Hinduism	231	85.56
Buddhism	29	10.74
Christianity	6	2.22
Others	4	1.48
**Type of Family**		
Single	152	56.3
Extended	118	43.7
**Occupation**		
Business	87	32.22
Agriculture	48	17.78
Governmental/private job	31	11.48
Housewife	31	11.48
Pension	31	11.48
Landlord	19	7.04
Teaching	11	4.07
Driving	5	1.85
Others	7	2.59

### Measures of physical activity

It was found that about 52% of the diabetic patients were moderately physically active (MET values ranging between 600 and 3000) and 28% were highly active (MET value equal to or greater than 3000) while about 20% were found to have a low physical activity level (MET value less than 600) (Table 2). Walking for travel was the biggest contributor as it contributed to almost half (46%) of the total physical activity. Work related activities were found to be the second major contributors (34%) to total physical activity. On the other hand, recreational activities (leisure activities) were found to be much less common (20% of the total physical activity) among the participants (Table 2).

**Table 2:** Different levels of physical activity with mean, SE and 95% CI of MET values among urban diabetic patients in Nepal (n = 270).

Level of physical activity	Frequency	Percentage	Mean	SE	95%CI	
**Low**	55	20.37	372.58	22.18	328.92	416.25
**Medium**	140	51.85	1554.00	57.43	1440.94	1667.06
**High**	75	27.78	6433.87	620.49	5212.24	7655.50
**Total**	**270**	**100**	**2668.86**	**226.61**	**2222.71**	**3115.01**

Female respondents were found to be more sedentary than men. The women had an average sedentary time of 307 minutes per day compared to 257 minutes per day among their male counterparts (p<0.05). The average MET score was around 2669, with an average ranging between 373 and 6434.

The respondents were divided into 4 different categories to see if they differed from each other in terms of the duration since their diabetes diagnosis (Table 3). The 4 categories developed were: 1 = those who were diagnosed with type-2 diabetes for less than 6 months (n = 7), 2 = those who were diagnosed between 6 months and <2 years (n = 32), 3 = those who were diagnosed between 2 years and <5 years (n = 77), and finally, 4 = those who were diagnosed between 5 years and more (n = 154).

**Table 3:** Comparison of MET values by Duration since diagnosis.

Row Mean- Col Mean	<6 months	6mths to <2 years	2 years to <5 years
**6mths to <2 years**	3534.91 (0.129)		
**2 years to <5 years**	1264.05 (1.000)	-2270.86 (0.021*)	
**5 years and more**	1303.9 (1.000)	-2231.01 (0.012*)	39.8442 (1.000)

*P<0.05

Upon comparing these four groups using post-hoc multiple comparisons with Bonferroni adjustment, it was seen that there was a difference between these groups in terms of their MET measures (Table 3). More specifically, those belonging to the groups 3 and 2 differed from each other (3 had lower MET than 2), and those belonging to groups 4 and 2 differed from each other (4 had lower MET than 2) (P<0.05).

It means that those who had been diagnosed with Type-2 diabetes between 6 months and less than 2 years had higher MET scores than those who had been diagnosed between 2 years and more. The group also had a higher MET score than among those diagnosed for less than 6 months. However, the result was not statistically significant. No such difference was observed between each other in the rest of the groups.

Multinomial logistic regression analysis of high and moderate level physical activity among urban diabetic patients in Nepal in Table 4 revealed that male diabetic patients were found to have higher likelihood for high physical activity but not for moderate level physical activity (High OR = 1.56; p>0.05; Moderate OR = 0.36; p>0.05) than their female counterparts. With regards to age, with every increasing year, the people living with diabetes were slightly less likely to be both moderately and highly active compared with having low level physical activity (OR = 0.98; p>0.05). It was also observed that with an increase in the level of education, the physical activity increased. The odds of moderate physical activity among those with primary and secondary education compared to those who were illiterate were 1.86 (p>0.05) and 1.90 (p>0.05) respectively, and for high physical activity were 1.97 (p>0.05) and 2.05 (p>0.05) respectively. The diabetics living in nuclear families were more likely to be moderately active but less likely to be highly active than those living in extended families (Moderate OR = 1.19; p>0.05; High OR = 0.73; p>0.05).

**Table 4:** Multinomial logistic regression analysis of high and moderate level physical activity among urban diabetic patients in Nepal (n = 270).

Characteristics	N	Unadjusted OR		Adjusted OR	
		Moderate level PA	High level PA	Moderate level PA	High level PA
**Gender**					
Female	103	1	1	1	1
Male	167	0.51 (0.26–1.00)	1.15 (0.54–2.46)	0.36 (0.12–1.02)	1.56 (0.49–4.92)
**Age**					
	270	1.00 (0.97–1.03)	1.01 (0.98–1.05)	0.98 (0.94–1.03)	0.98 (0.93–1.03)
**Education**					
Illiterate	33	1	1	1	1
Primary	68	1.50 (0.54–4.20)	1.78 (0.52–6.02)	1.86 (0.43–7.97)	1.97 (0.35–11.00)
Secondary and above	169	1.37 (0.55–4.20)	1.95 (0.66–5.74)	1.90 (0.44–8.09)	2.05 (0.38–10.79)
**Type of family**					
Extended	118	1	1	1	1
Single	152	1.54 (0.84–2.97)	0.93 (0.46–1.88)	1.19 (0.54–2.60)	0.73 (0.30–1.75)

The analyses were adjusted for age, sex, education, place of residence, income, religion, caste, marital status, occupation and type of family

### Source of encouragement

Doctors were the most important influencing change agents who motivated the diabetic patients to become physically active. Out of total respondents, 223 were involved in act of physical activity, about half (108/223, 48%) of the respondents said that they felt encouraged to be physically active by doctors, followed by family and friends (64/223, 29%). Self-motivation (37/223, 17%) and health-related newspaper information (14/223, 6%) also made them feel driven to remain physically active. Likewise, 63% (171/270) respondents believed that they were personally living at risk of diabetic complications, and 79% (214/270) respondents believed that there could be negative consequences of diabetes in general.

### Facilitators of physical activity

In general, most respondents either agreed or strongly agreed with most benefit items in the EBBS scale (results expressed in terms of mean±SD) (Table 5). The item “Exercising helps me sleep better at night” amounted to strong agreement (3.59±0.66). Other benefit item scores approached “agree” option of the scale, for example: “My disposition is improved with exercise” (2.97±0.50), “Exercise helps me decrease fatigue” (2.79±0.88), “Exercising increases my acceptance by others” (2.91±0.58), “I will prevent heart attacks by exercising” (2.93±0.87), and “I will live longer if I exercise” (2.91±0.95). None of the respondents completely disagreed with any of the benefit statements. When those responses were stratified by gender, the opinions were found to differ on average. In some items of benefit subscale like “I will prevent heart attacks by exercising” (male = 2.14±0.88 and female = 2.71±0.94), “Exercising will keep me from having high blood pressure” (male = 2.10±0.80 and female = 2.70±0.96), and “I will live longer if I exercise” (male = 1.98±0.91 and female = 2.43±0.99), males tended to lean more towards disagreement while females either tended towards neutrality or agreed to the items. Meanwhile, both groups strongly agreed that “Exercise helps them sleep better at night” (male = 3.62±0.61 and female = 3.51±0.72). In summary, it was found that almost all respondents responded to the facilitator items with an average item score of two or above.

**Table 5:** Perceived exercise benefit sub-scales among urban female and male diabetic patients in Nepal (n = 270).

Perceived Benefit Items	Total	Female	Male
Life enhancement	Mean(SD)	Mean(SD)	Mean(SD)
25. My disposition is improved with exercise	2.97(0.50)	2.86(0.50)	3.035(0.48)
26. Exercising helps me sleep better at night	3.59(0.66)	3.51(0.72)	3.62(0.61)
29. Exercise helps me decrease fatigue	2.79(0.88)	2.59(0.89)	2.90(0.85)
32. Exercising improves my self-concept	3.15(0.68)	2.97(0.66)	3.25(0.66)
34. Exercising increases my mental alertness	3.15(0.60)	2.96(0.64)	3.26(0.55)
35. Exercise allows me to carry out normal activities without becoming tired	3.07(0.81)	2.79(0.92)	3.24(0.68)
36. Exercise improves the quality of my work	3.03(0.73)	2.78(0.73)	3.17(0.69)
41. Exercise improves overall body functioning for me	3.33(0.64)	3.10(0.67)	3.47(0.57)
**Physical Performance**			
7. Exercise increases my muscle strength	3.24(0.63)	3.04(0.67)	3.35(0.58)
15. Exercising increases my level of physical fitness	3.33(0.59)	3.07(0.62)	3.49(0.59)
17. My muscle tone is improved with exercise	3.23(0.63)	3.03(0.62)	3.34(0.61)
18. Exercising improves functioning of my cardiovascular system	3.35(0.61)	3.15(0.62)	3.47(0.56)
22. Exercise increases my stamina	3.21(0.66)	3.00(0.69)	3.33(0.60)
23. Exercise improves my flexibility	3.31(0.66)	3.20(0.69)	3.37(0.62)
31. My physical endurance is improved by exercising	3.25(0.59)	3.02(0.63)	3.25(0.65)
43. Exercise improves the way my body looks	3.11(0.71)	2.88(0.73)	3.25(0.65)
**Psychological outlook**			
1. I enjoy exercise	3.35(0.70)	3.15(0.73)	3.47(0.64)
2. Exercise decreases feelings of stress and tension for me	3.35(0.68)	3.15(0.68)	3.47(0.64)
3. Exercise improves my mental health	3.30(0.70)	3.11(0.74)	3.41(0.64)
8. Exercise gives me a sense of personal accomplishment	3.41(0.64)	3.24(0.67)	3.50(0.58)
10. Exercising makes me feel relaxed	3.49(0.64)	3.39(0.64)	3.53(0.62)
20. I have improved feelings of well-being from exercise	3.33(0.74)	3.17(0.84)	3.43(0.64)
**Social Interaction**			
11. Exercising lets me have contact with friends and persons I enjoy	3.13(0.85)	3.05(0.88)	3.17(0.83)
30. Exercising is a good way for me to meet new people	3.15(0.76)	2.96(0.77)	3.26(0.72)
38. Exercise is good entertainment for me	2.86(0.91)	2.68(0.91)	2.96(0.89)
39. Exercising increases my acceptance by other	2.91(0.58)	2.73(0.57)	3.02(0.55)
**Preventive Health**			
5. I will prevent heart attacks by exercising	2.93(0.87)	2.71(0.94)	2.14(0.88)
13. Exercising will keep me from having high blood pressure	3.20(0.75)	2.70(0.96)	2.10(0.80)
27. I will live longer if I exercise	2.91(0.95)	2.43(0.99)	1.98(0.91)

### Barriers to physical activity

Most respondents either disagreed or strongly disagreed with the barrier items (Table 6). There were even clear-cut disagreements in response to some barrier items. For example: “My spouse (or significant other) does not encourage exercising” (1.33±0.80), “My family members do not encourage me to exercise” (1.40±0.66), “I am too embarrassed to exercise” (1.44±0.74), and “It costs too much to exercise” (1.46±0.62) reflected clear disagreement indicating that these items did not reflect the respondents’ perceived barriers.

**Table 6:** Perceived physical activity barrier sub-scales among urban female and male diabetic patients in Nepal (n = 270).

Perceived Barrier Items	Total	Female	Male
Exercise Milieu	Mean(SD)	Mean(SD)	Mean(SD)
9. Places for me to exercise are too far away	2.04(0.82)	2.23(0.88)	1.92(0.76)
12. I am too embarrassed to exercise	1.44(0.74)	1.64(0.86)	1.31(0.63)
14. It costs too much to exercise	1.46(0.62)	1.47(0.65)	1.44(0.60)
16. Exercise facilities do not have convenient schedule for me	2.44(0.99)	2.65(1.02)	2.31(0.94)
28. I think people in exercise cloths look funny	1.67(0.74)	1.73(0.75)	1.63(0.73)
42. There are too few places for me to exercise	2.13(0.88)	2.12(0.90)	2.13(0.87)
**Time expenditure**			
4. Exercising takes too much of my time	2.42(0.92)	2.51(0.93)	2.36(0.90)
24. Exercise takes too much time from family relationships	2.19(0.93)	2.51(1.74)	1.98(0.77)
37. Exercise takes too much time from my family responsibilities	2.09(0.92)	2.36(1.05)	1.91(0.77)
**Physical exertion**			
6. Exercise tires me	2.37(0.95)	2.71(0.94)	2.14(0.88)
19. I am fatigued by exercise	2.34(0.92)	2.70(0.96)	2.10(0.80)
40. Exercise is hard work for me	2.16(0.97)	2.43(0.99)	1.98(0.91)
**Family discouragement**			
21. My spouse (or significant other) does not encourage exercising	1.33(0.80)	1.19(0.86)	1.41(0.75)
33. My family members do not encourage me to exercise	1.40(0.66)	1.40(0.67)	1.39(0.64)

However, there were some barrier items which scored higher than others. For example, “Exercise facilities do not have convenient schedule for me” (2.44±0.99), and Exercising takes too much of my time” (2.42±0.92). Further gender-stratified analysis revealed that some items scored higher for women than for men. For example: Exercise takes too much of my time (2.51±0.93), Exercise tires me (2.71±0.94) and I am fatigued by exercise (2.70±0.96) were found to matter more to women than men.

Multinomial logistic regression analyses were conducted to find out which factors acted upon moderate level and high level physical activity as facilitators and barriers (Table 7). The analyses revealed that respondents were nearly 3.5 times more likely to have moderate physical activity (OR = 3.49, CI: 1.60–7.64) and 3.77 times more likely to have high physical activity (OR = 3.77, CI: 1.56–9.03) with every unit increase in the measure of perception that physical activity enhances one’s physical performance (improves body look, increases muscle strength, leads to better physical fitness, improves muscle tone and flexibility. Likewise, the respondents were 1.96 times more likely to have moderate physical activity (OR = 1.96, CI: 1.04–3.71) with the benefit perception that physical activity enhances psychological outlook (decreases the feelings of stress, improves mental health and gives the feeling of relaxation) and 2.26 times more likely to have high physical activity (OR = 2.26, CI: 1.09–4.69). Likewise, the diabetes patients were 1.73 times more likely to have moderate physical activity (OR = 1.73, CI: 1.05–2.85) and 1.78 times more likely to have high physical activity (OR = 1.78, CI: 1.02–3.11) with the benefit perception that physical activity acts as a means of preventive health. As for social interaction, those with the perception that physical activity provides opportunity to interact with others were 2.28 times more likely to have moderate physical activity (OR = 2.28, CI: 1.25–4.15) and 1.54 times more likely to have high physical activity (OR = 1.54, CI: 0.80–2.97).

**Table 7:** Multinomial logistic regression analysis of barriers and facilitators of physical activity among urban diabetic patients in Nepal (n = 270).

Level of Physical activity	Unadjusted OR	Adjusted		
		OR	P-value	[95% Conf. Interval]
**1) Moderate physical activity**^1^				
**a) Perceived Benefits**				
Psychological Outlook	1.99	1.96	0.04*	1.04–3.71
Preventive Health	1.51	1.73	0.03*	1.05–2.85
Physical Performance	1.97	3.49	0.002*	1.60–7.64
Social Interaction	2.10	2.28	0.007*	1.25–4.15
Life Enhancement	2.13	2.21	0.04*	1.02–4.80
**b) Perceived Barriers**				
Time Expenditure	0.68	0.67	0.08	0.43–1.05
Physical Exertion	1.11	0.89	0.60	0.59–1.36
Exercise Milieu	1.41	1.12	0.74	0.55–2.39
Family Discouragement	0.47	0.66	0.13	0.38–1.14
**2) High physical activity**^1^				
**a) Perceived Benefits**				
Psychological Outlook	3.53	2.26	0.03*	1.09–4.69
Preventive Health	2.22	1.78	0.04*	1.02–3.11
Physical Performance	3.95	3.77	0.003*	1.56–9.03
Social Interaction	3.06	1.54	0.19	0.80–2.97
Life Enhancement	4.97	2.81	0.02*	1.17–6.76
**b) Perceived Barriers**				
Time Expenditure	0.54	0.62	0.07	0.37–1.04
Physical Exertion	0.71	0.69	0.12	0.42–1.11
Exercise Milieu	1.29	1.28	0.53	0.58–2.83
Family Discouragement	0.48	0.69	0.22	0.38–1.25

^1^All analyses made with reference to low physical activity level

*P<0.05

Multinomial logistic regression analyses were also conducted to reveal the factors that influenced moderate and high level physical activity with a unit increase in the measure of the potential barriers separately (Table 7). The analysis revealed that with the barrier perception of time expenditure not allowing the respondents to be physically active, the odds of having moderate physical activity level were 0.67 and of high physical activity level were 0.62 (p>0.05). With the perception that the physical exertion during physical activity is a barrier, the odds of moderate activity were down to 0.89 and those of high physical activity to 0.69 (p>0.05). Conversely, when the immediate living environment acted as a barrier to being physically active, the diabetics were 1.12 times more likely to have moderate physical activity and 1.28 times more likely to have high physical activity (p>0.05). Finally, with the feeling that family or spouse discouraged them from being physically active, the odds of physical activity were found to decrease the odds of being moderately physically active were 0.66 and of having high physical activity were 0.69 (p>0.05).

## Discussion

This cross-sectional study conducted among urban diabetic patients from Nepal found that they were fairly physically active. Slightly more than half (140/270, 52%) of the total diabetic respondents were moderately physically active. Walking for travel and work-related activities were the major contributors in their physical activity. Almost half of the total physical activity, i.e. 46% of moderate physical activity and 15% of high physical activity were because of walking alone. Doctors were the main motivators to encourage the respondents towards remaining physically active. The analysis of facilitators and barriers for different levels of physical activity showed that perceived physical fitness, strength and flexibility, better sleep at night, social interaction and longevity were the major facilitators for physical activity. On the other hand, family responsibilities, busy schedule and family discouragement were the common barriers to being physically active.

### Prevalence of physical activity

This study found that diabetic patients living in urban areas of two cities of Nepal were fairly physically active. Slightly more than half (52%) of the diabetic respondents were moderately physically active. In a similar South Asian study, majority of the diabetic patients (86%) from Sri Lanka were found to be physically active [25]. Similarly, two Iran based studies also found that moderate physical activity among the diabetic patients was fairly high ranging from 57.5% to 73% [26, 27]. This fairly good level of physical activity among urban diabetic patients in Nepal could possibly be explained by a good level of awareness on the advantages of physical activity in glucose regulation and the fear of negative consequences of diabetes. Seventy-nine percent of respondents in the study believed that there could be negative consequences of diabetes. Likewise, 63% of the respondents believed that they were personally living at risk of further consequences because of diabetes. These factors could have acted as reinforcements in the respondents approach towards physical activity. The respondents in this study had an overall good education and spending capacity and thus represented a fairly better-off section of the population who could afford to attend private clinics for diabetic management. Previous studies have also shown that people with good education and better socioeconomic status are more likely to be physically active [26].

With an increase in age, a slight decline in physical activity was observed. This is in line with similar findings from previous studies [6, 28]. As people age, many start suffering from multiple diseases including chronic diseases. Co-morbidities are known to be major barriers to adopting physical activity among diabetic patients [29–31]. Male diabetic patients were found to have had slightly higher likelihood of physical activity than their female counterparts. Although the difference did not amount to statistical significance, this higher level of physical activity among males could be due to the culture of involvement among males in outdoor activities which are more vigorous than indoor activities performed by women. In Nepal, women are still usually expected to do household work and support their family and therefore are more occupied inside their households [17]. Another study found that mobility rate of Nepalese males was three times higher than that of females [19].

It was observed that the individuals diagnosed with diabetes between 6 months to less than 2 years ago were found to have attained higher MET values than those who had been diagnosed with diabetes either 6 months before the study or for more than 2 years at the time of survey. To explain this finding, it could be argued that those individuals who are in the initial months since being diagnosed take some time to come to terms with the new disease and are possibly still facing the struggle to adjust to a new lifestyle. After the initial 6 months, it could be argued that they come to terms with their new conditions and decide to take control of them and adopt a healthier lifestyle. This desire to prevent further aggravation of their health might contribute to them becoming more physically active. However, as time passes, they get used to the new disease and slack in terms of physical activity.

Contrary to our findings, people have been found to be more physically active in the initial days of diabetes diagnosis [21]. However, it is to be taken into consideration that we have considered 6 months as the immediate period since diagnosis and the time between 6 months to 1 year as a longer timeframe since diagnosis unlike in the study by Plotnikoff RC, et al. where they considered less than one year, and more than one year as the minimum timeframe since the time of diagnosis.

### Components of physical activity

Travel (46%) and work related activities (34%) were found to be the major contributors towards physical activity in the present study. Walking is the best means of remaining physically active as it relatively easy and does not cost anything. People endorsed walking either as a part of their planned exercise, a leisure activity or as a means of transportation. Furthermore, in Nepal like in many other developing countries, physical activity relates to the work people do in their daily lives. They do not engage in other specific sets of activities just for the sake of being physically active. All the household chores, agriculture-related field work and other occupation-related activities are what comprise most of physical activity in these contexts [20, 32, 33]. Instead of focusing only on structured physical activity, giving more emphasis to lifestyle physical activity is known to be more beneficial (and probably more practical) for diabetic patients. Activities like jogging, walking, tai-chi, yoga and gardening have been recommended [34].

Leisure activity participation in this study was found to be lower, (20% only), compared to other domains. Even within this 20%, 15% was because of walking alone. Mostly Nepalese adults in urban areas spend their leisure time watching television, chatting around or sleeping instead of doing physically-demanding outdoor activities [20]. These activities contribute towards people’s entertainment and socialization. It might be also because Nepal does not have widely-accessible public exercise amenities (for example: public parks with different exercise equipments). Nepal does not yet have a culture of doing outdoor activities for physical fitness except for some young people involved in sports. The private gyms and fitness amenities that are available in some places within urban areas are in limited number and they do not yet have a wider acceptance as places to pursue physical activity due to cultural indifference to physical activity as a deliberate action done at leisure time out of choice alone. On the contrary, in most high-income countries those facilities are made readily available by the local councils or municipalities to be used by the general public. They also have a higher acceptance rate as go-to places for being physically active in those countries. Likewise, there is a more widespread awareness in general on the importance of being physically active for good health and wellbeing in all age groups. This could potentially explain the result that the contribution of vigorous intensity leisure time physical activity to total MET-minutes/week for the populations from high income countries like Australia and USA was more than 50% and it was more than 45% in New Zealand and Canada [35].

### Source of encouragement

Doctors were the main motivators who encouraged the respondents towards remaining physically active in our study. Most of them were found to have increased their physical activity after the diagnosis of diabetes. In previous studies, it was observed that South Asian patients consider doctors as the authoritative source of knowledge on diabetes [29, 36, 37]. Almost half (48%) of the respondents in our study had started doing physical activity after their doctor’s recommendation. They may have received counseling from health personnel to increase their physical activity to live long and stay healthy. The diagnosis may have also helped to highlight the worsening condition in absence of further intervention [38, 39]. In addition, people usually have warning signs or pre-diabetes symptoms prior to their diagnosis that could heighten their awareness for the necessity of behavior change [40]. By contrast however, a study reported that health professionals were reportedly not good enough in providing adequate information about the importance of physical activity or about its duration and frequency. Consequently, patients were less motivated for physical activity [41].

### Facilitators and barriers to physical activity

The analysis of facilitators and barriers for different levels of physical activity showed that the factors that encouraged them for physical activity were more positively perceived by most diabetic patients than were the factors that barred them from being physically active. Physical fitness, strength and flexibility, better sleep at night, social interaction and longevity were found to amount as the major facilitators of physical activity. On the other hand, family responsibilities, busy schedule and family discouragement were reported as the common barriers to being physically active.

#### Facilitators

The highest scorer of perceived benefit item encompasses multiple health aspects like fitness, stamina, flexibility, strength, muscle tone and physical appearance. This preference for physical performance behind being physically active could potentially be explained by the reason that people want to do any kind of physical activity to feel relaxed, to look good and to increase their stamina. Similar to our finding, Mexican, French and other Asian diabetic patients in other studies had also mentioned that they were physically active because they wanted to look good, feel more rested and at the same time to have more energy [42–45]. Although the perception of “zero-sized figure” as beautiful is not common in Nepal, not at least so especially among the middle ages like the respondents in this study, very big body is not preferred either, unlike as demonstrated in a Bangladeshi study where big body was equated with health [43]. In our study respondents from these age groups were found to be more concerned about increasing their stamina and fitness through physical activity.

One of the main concerns behind being physically active among the respondents in this study was getting better sleep at night, decrease fatigue and increase mental alertness. The respondents reported having experienced better sleep following physical activity. Different epidemiological studies have found that different levels of physical activity have varying degrees of positive effect on sleep-related indicators [46, 47]. On the other hand, better sleep helps to reduce the insulin resistance [48, 49]. Reduction in anxiety symptoms, increased metabolism, changes in body temperature, and resetting the sleep wake cycle have also been suggested as some reasons behind positive linkage between sleep and physical activity [47].

Another reason that kept the diabetic patients physically active was a feeling of decreased fatigue. Regular physical activity has been known to improve aerobic capacity (body’s ability to take in and use oxygen) and muscle mass. With the improvement in body’s ability to transport and use oxygen, regular daily activities become easier to perform with less fatigue. Physical activity also increases metabolism and leads to mood elevation, which are all believed to contribute to decreased fatigue among people living with type-2 diabetes [50]. Mental alertness was another reason cited by the diabetic patients behind their motivation for physical activity. It has been known that brain regions involved in memory function are stimulated by exercise to release brain-derived neurotrophic factor (BDNF), which is known to rewire memory circuits and make them work better [51].

The motivation among participants in the present study to do more physical activity was found to be influenced by their peers. This could be because people might get encouraged if they have company while going for a walk, or any other form of deliberate physical exercise aimed at health and wellbeing. They might also get inspired by listening to the peer’s success stories of having reduce or at least maintained low blood glucose level through different modes of physical activity. Chlebowy et al also reported that social support was associated with increased adherence to physical activity among type 2 diabetic patients [52]. The reason participants stated was that going to the gym with group made the job easier than engaging in physical activity alone. Participants expressed excitement and happiness about meeting their friends while doing such activities. Lack of social support, on the other hand, was reported affecting engagement in physical activity negatively.

In the present study, participants also reported that they felt their blood glucose level to have fallen following being physically active and this further motivated them to be more actively involved. They perceived it as a good investment in their body and this finding is also supported by other studies [29, 53, 54]. The possible explanation to this phenomenon is that the feeling of personal accomplishment through actual or perceived feeling of regulated blood glucose level increases one's self-efficacy towards physical activity and encourages them for further activity.

#### Barriers

Time expenditure was one of the perceived barriers to physical activity among the diabetic patients in this study where family responsibility was the major concern. However, it was not statistically significant (Table 6). The respondents planned their time around family and work and therefore did not feel they had any extra time left for physical activity. The study also found low physical activity among women compared to men. Nepali women in particular are more obliged towards family responsibilities than men and therefore stay indoors attending to those obligations. Women are also expected to prioritize their obligations to kin over the pursuit of their own interests and activities. Among men, the family responsibilities of helping kids do their homework, taking children to school or just being engaged in other works at home could be some of the deterrents. Similar to this study findings, diabetic patients in UAE [55] and Denmark [30] also found time and family responsibilities as a potential barrier. However, the interesting fact is that none of the participants from present study reported that they completely stopped doing physical activity. They somehow managed their time and remained physically active. It is also noteworthy that the household work is itself a part of physical activity.

The role of family members has been found to be important in promoting physical activity not only as a supportive factor but also as a critical motivator. The respondents in the present study reported family discouragement as a perceived barrier to physical activity though the finding was not statistically significant. The finding of this factor being considered a perceived barrier aligned with that from some other studies [31, 45, 52]. If the family members support and encourage people with diabetes, their uptake of physical activity could increase spontaneously.

Surprisingly, unlike in a previous study factors listed within the domain of exercise milieu like exercise locations being far away, the feeling of embarrassment towards exercising and high costs of exercise were not perceived as barriers to physical activity in our study [55]. Most people in the present study practiced walking or other daily activities embedded in their household chores for physical activity, so the issues of exercise locations being far away or having a high cost or inconvenient schedule were not relevant for these respondents. One possible reason might be that, in Nepal, attending gyms for physical fitness or subscribing to specific exercise courses is an unfamiliar and new concept, especially among the older respondents. The feeling of embarrassment towards exercising did not amount to a potential barrier, nor did the issue of wearing specific garments while performing exercise. In contrast, it has been observed in other studies that the fear of discrimination or judgments from other groups for not obliging to specific clothes for the physical activity in question could also deter people from being physically active [54]. As observed in this study, the respondents usually wore informal clothing in which they were comfortable, which were not necessarily exercise gears, and never felt embarrassed nor did they complain about those garments. Especially women are known to feel embarrassed to go outdoors in informal outfits in front of men in some cultures [29]. The present study findings did not find such gender-specific differences either. Regardless of gender, the study respondents expressed that they were free to move.

To conclude on barriers, although family responsibilities, busy schedule and family discouragement were found to score more than other factors as barriers to physical activity, it was found that none of those barrier variables amounted to statistical significance.

### Implication

With this knowledge of what seems to promote physical activity and what seems to hinder it, the next stage in research on physical activity in Nepal could focus at intervention research. There have almost been no initiatives made in Nepal of interventional research on physical activity, let alone in the area of diabetes prevention or management. So, setting up of a tailor-made intervention to see the effectiveness of physical activity in diabetes prevention, and complication reduction and management could further this research agenda ahead.

Walking has been reported as the commonest physical activity. Therefore, walking lanes and open spaces should be preserved and made safe for walking, so that people won’t be deterred from this practice.

Future research should also explore what other ways of remaining physically active could be of interest to a wider range of urban-residing diabetic patients in Nepal. This will help cater to the needs of the urban residents, not only those living with diabetes in particular but also all urban residents in general.

### Strengths and limitations

Since there are very few published studies in Nepal about physical activity among diabetic patients, this study contributes to the literature and could potentially help further the research in physical activity among diabetics. However, the study does have some shortcomings.

As a cross-sectional study, our study cannot imply causality regarding physical activity facilitators and barriers among the urban diabetic patients. Self-reported measures could have overestimated the respondents’ physical activity levels. Self-reported measures could have overestimated the respondents’ physical activity levels. MET values of some activities, which involve high breathing, have not been derived from actual oxygen consumption, so the risk of overestimation could not be ruled out.

The information collected in minutes of physical activity might also have been incorrectly reported by some individuals due to the inherent difficulty of recalling the amount of minutes spent in one week doing any particular activity.

Another limitation is that EBBS had not been validated for use in Nepal. We collected information from only the individuals of certain socio-economic gradient who could afford healthcare at private health institution, so our respondents were not representative of all the urban-residing diabetic patients. So, there is limited generalizability of our findings.

## Conclusion

The present study estimated the prevalence of physical activity among diabetic patients residing in urban regions of Nepal and explored the factors that facilitate or hinder physical activity among them. More than half of the study participants were found to be moderately active and about one third were highly physically active. Moderate intensity activities and walking in particular, comprised most of the total physical activity, which meant that vigorous intensity activities had a limited share. Despite the fact that milieu barrier were not found to significantly hinder people’s physical activity, it should be noted that due to rapid urbanization and motorization, less and less open areas are being available in cities. Additionally, urban air pollution is steadily on a rise in Nepal and it has been recommended not to walk outdoors in the morning in these areas due to high concentration of particulate matter (PM_2.5_) in the air, especially during winter months. Therefore, future research should explore what other ways of remaining physically active apart from walking and doing household chores could be of interest to a wider range of urban-residing diabetic patients in Nepal. Especially important is the idea of promotion of suitable leisure time physical activities, which seem to be very low among Nepali population in general as demonstrated in other studies and also observed in this study. Such innovative double-pronged approaches that promote the facilitators and address the barriers of physical activity can have positive results through prevention of complications and can help reduce the healthcare costs of complication management. Health worker’s role in promoting more diversified activities that could fit within people’s everyday activities could prove important since their recommendation was found to be valuable and people seemed to take their suggestions of being physically active seriously.

## Abbreviations

EBBS: Exercise Barriers and Benefits Survey
GPAQ: Global Physical Activity Questionnaire
HbA1c: Glycated haemoglobin
KM: Kilometer
MET: Metabolic Equivalent
NCD: Non-Communicable Diseases
NDA: Nepal Diabetes Association
NRs: Nepalese Rupees
OPD: Out Patient Department
OR: Odds ratio
PA: Physical Activity
PP: Postprandial
STEPS: STEPwise approach to Surveillance
WHO: World Health Organization

## References

[1] International Diabetes Federation. IDF DIABETES ATLAS. United Kingdom: 2017.

[2] World Health Organization. Diabetes Factsheet. Geneva, Switzerland WHO, 2016.

[3] World Health Organization. NCD Country Profile. Geneva, Switzerland WHO, 2014.

[4] International Diabetes Federation. Nepal: International Diabetes Federation. https://www.idf.org/our-network/regions-members/south-east-asia/members/97-nepal.html.

[5] Nepal Diabetes Association. The prevalence of diabetes in the people 20 years and above. Nepal: Nepal Diabetes Association, 2016.

[6] Thomas N, Alder E, Leese GP. Barriers to physical activity in patients with diabetes. Postgraduate medical journal. 2004;80(943):287–91. Epub 2004/05/13. doi: 10.1136/pgmj.2003.010553 . 15138320 10.1136/pgmj.2003.010553 PMC1742997

[7] Helmrich SP, Ragland DR, Leung RW, Paffenbarger RSJ. Physical Activity and Reduced Occurrence of Non-Insulin-Dependent Diabetes Mellitus. N Engl J Med. 1991;325(3):147–52. doi: 10.1056/NEJM199107183250302 . 2052059 10.1056/NEJM199107183250302

[8] Colberg SR, Sigal RJ, Fernhall B, Regensteiner JG, Blissmer BJ, Rubin RR, et al Exercise and Type 2 Diabetes: The American College of Sports Medicine and the American Diabetes Association: joint position statement. Diabetes care. 2010;33(12):e147–67. doi: 10.2337/dc10-9990 . 21115758 10.2337/dc10-9990 PMC2992225

[9] Sluik D, Buijsse B, Muckelbauer R, Kaaks R, Teucher B, Johnsen NF, et al Physical Activity and Mortality in Individuals With Diabetes Mellitus: A Prospective Study and Meta-analysis. Archives of internal medicine. 2012;172(17):1285–95. Epub 2012/08/08. doi: 10.1001/archinternmed.2012.3130 . 22868663 10.1001/archinternmed.2012.3130

[10] World Health Organization. Physical activity factsheet. Geneva, Switzerland: WHO, 2016.

[11] World Health Organization. Global Strategy on Diet, Physical Activity and Health: WHO. http://www.who.int/dietphysicalactivity/pa/en/.

[12] Duclos M, Coudeyre E, Ouchchane L. General practitioners' barriers to physical activity negatively influence type 2 diabetic patients' involvement in regular physical activity. Diabetes care. 2011;34(7):e122 Epub 2011/06/29. doi: 10.2337/dc11-0140 . 21709288 10.2337/dc11-0140 PMC3120192

[13] Umpierre D, Ribeiro PA, Kramer CK, Leitao CB, Zucatti AT, Azevedo MJ, et al Physical activity advice only or structured exercise training and association with HbA1c levels in type 2 diabetes: a systematic review and meta-analysis. Jama. 2011;305(17):1790–9. Epub 2011/05/05. doi: 10.1001/jama.2011.576 . 21540423 10.1001/jama.2011.576

[14] American Diabetes Association. Standards of Medical Care in Diabetes—2016. Diabtes Care. 2016;39(suppl 1):s27. 15618112

[15] Aryal K, Neupane S, Mehata S, Vaidya A, Singh S, Paulin F, et al. Non Communicable Diseases Risk Factors: STEPS Survey Nepal 2013. Kathmandu, Nepal: Nepal Health Research Council.

[16] Macniven R, Bauman A, Abouzeid M. A review of population-based prevalence studies of physical activity in adults in the Asia-Pacific region. BMC public health. 2012;12:41 Epub 2012/01/19. doi: 10.1186/1471-2458-12-41 . 22251660 10.1186/1471-2458-12-41 PMC3293715

[17] Parajuli J, Saleh F, Thapa N, Ali L. Factors associated with nonadherence to diet and physical activity among Nepalese type 2 diabetes patients; a cross sectional study. BMC research notes. 2014;7:758 Epub 2014/10/26. doi: 10.1186/1756-0500-7-758 . 25344089 10.1186/1756-0500-7-758 PMC4230343

[18] Ghimire Saruna. Barriers to Diet and Exercise among Nepalese Type 2 Diabetic Patients. International Scholarly Research Notices, 2017;2017:9. 10.1155/2017/1273084 PMC5733940 29349287

[19] Vaidya A, Krettek A. Physical activity level and its sociodemographic correlates in a peri-urban Nepalese population: a cross-sectional study from the Jhaukhel-Duwakot health demographic surveillance site. The international journal of behavioral nutrition and physical activity. 2014;11(1):39 Epub 2014/03/19. doi: 10.1186/1479-5868-11-39 . 24628997 10.1186/1479-5868-11-39 PMC3984675

[20] Panter-Brick C. The energy cost of common tasks in rural Nepal: levels of energy expenditure compatible with sustained physical activity. European journal of applied physiology and occupational physiology. 1992;64(5):477–84. Epub 1992/01/01. . 1612091 10.1007/BF00625071

[21] Plotnikoff RC, Lippke S, Karunamuni N, Eves N, Courneya KS, Sigal R, et al Co-morbidity, functionality and time since diagnosis as predictors of physical activity in individuals with type 1 or type 2 diabetes. Diabetes research and clinical practice. 2007;78(1):115–22. Epub 2007/03/24. doi: 10.1016/j.diabres.2007.02.016 . 17379349 10.1016/j.diabres.2007.02.016

[22] World Health Organization. Global Physical Activity Questionnaire (GPAQ) Analysis Guide Geneva, Switzerland: WHO.

[23] Sechrist KR, Walker SN, Pender NJ. Development and psychometric evaluation of the exercise benefits/barriers scale. Research in nursing & health. 1987;10(6):357–65. Epub 1987/12/01. . 3423307 10.1002/nur.4770100603

[24] Creative Research Systems. Sample Size Formulas 2016. https://www.surveysystem.com/sample-size-formula.htm.

[25] Ranasinghe DC, Ranasinghe P, Jayawardena R, Matthews DR, Katulanda P. Evaluation of physical activity among adults with diabetes mellitus from Sri Lanka. International archives of medicine. 2014;7:15 Epub 2014/04/09. doi: 10.1186/1755-7682-7-15 . 24708737 10.1186/1755-7682-7-15 PMC3996511

[26] Fattahi A., Barati M., Bashirian S., Moghadam RH. Physical Activity and Its Related Factors Among Type 2 Diabetic Patients in Hamadan. Iranian Journal of Diabetes and Obesity. 2014;6(2).

[27] Halali F, Mahdavi R, Asghari Jafarabadi M, Mobasseri M, Namazi N. A cross-sectional study of barriers to physical activity among overweight and obese patients with type 2 diabetes in Iran. Health & social care in the community. 2016;24(5):e92–e100. Epub 2015/06/24. doi: 10.1111/hsc.12263 . 26099926 10.1111/hsc.12263

[28] Nelson KM, Reiber G, Boyko EJ. Diet and exercise among adults with type 2 diabetes: findings from the third national health and nutrition examination survey (NHANES III). Diabetes care. 2002;25(10):1722–8. Epub 2002/09/28. . 12351468 10.2337/diacare.25.10.1722

[29] Lawton J, Ahmad N, Hanna L, Douglas M, Hallowell N. 'I can't do any serious exercise': barriers to physical activity amongst people of Pakistani and Indian origin with Type 2 diabetes. Health education research. 2006;21(1):43–54. Epub 2005/06/16. doi: 10.1093/her/cyh042 . 15955792 10.1093/her/cyh042

[30] Lidegaard LP, Schwennesen N, Willaing I, Faerch K. Barriers to and motivators for physical activity among people with Type 2 diabetes: patients' perspectives. Diabetic medicine: a journal of the British Diabetic Association. 2016;33(12):1677–85. Epub 2016/06/10. doi: 10.1111/dme.13167 . 27279343 10.1111/dme.13167

[31] Song M, Lee M, Shim B. Barriers to and facilitators of self-management adherence in Korean older adults with type 2 diabetes. International journal of older people nursing. 2010;5(3):211–8. Epub 2010/10/12. doi: 10.1111/j.1748-3743.2009.00189.x . 20925703 10.1111/j.1748-3743.2009.00189.x

[32] Bauman AE, Reis RS, Sallis JF, Wells JC, Loos RJ, Martin BW. Correlates of physical activity: why are some people physically active and others not? Lancet. 2012;380(9838):258–71. Epub 2012/07/24. doi: 10.1016/S0140-6736(12)60735-1 . 22818938 10.1016/S0140-6736(12)60735-1

[33] Panter-Brick C, Lotstein DS, Ellison PT. Seasonality of reproductive function and weight loss in rural Nepali women. Human reproduction (Oxford, England). 1993;8(5):684–90. Epub 1993/05/01. . 8314959 10.1093/oxfordjournals.humrep.a138120

[34] Pai LW, Chang PY, Chen W, Hwu YJ, Lai CH. The effectiveness of physical leisure time activities on glycaemic control in adult patients with diabetes type 2: A Systematic Review. JBI library of systematic reviews. 2012;10(42 Suppl):1–20. Epub 2012/01/01. doi: 10.11124/jbisrir-2012-251 . 27820154 10.11124/jbisrir-2012-251

[35] Bauman A, Bull F, Chey T, Craig CL, Ainsworth BE, Sallis JF, et al The International Prevalence Study on Physical Activity: results from 20 countries. The international journal of behavioral nutrition and physical activity. 2009;6:21 Epub 2009/04/02. doi: 10.1186/1479-5868-6-21 . 19335883 10.1186/1479-5868-6-21 PMC2674408

[36] Patel M, Patel IM, Patel YM, Rathi SK. Factors associated with consumption of diabetic diet among type 2 diabetic subjects from Ahmedabad, Western India. Journal of health, population, and nutrition. 2012;30(4):447–55. Epub 2013/01/12. . 23304911 10.3329/jhpn.v30i4.13328 PMC3763616

[37] Rhodes P, Nocon A. A problem of communication? Diabetes care among Bangladeshi people in Bradford. Health & social care in the community. 2003;11(1):45–54. Epub 2003/11/25. . 14629232 10.1046/j.1365-2524.2003.00398.x

[38] Schneider KL, Andrews C, Hovey KM, Seguin RA, Manini T, Lamonte MJ, et al Change in physical activity after a diabetes diagnosis: opportunity for intervention. Medicine and science in sports and exercise. 2014;46(1):84–91. doi: 10.1249/MSS.0b013e3182a33010 23860414 10.1249/MSS.0b013e3182a33010 PMC4028702

[39] Donahue KE, Mielenz TJ, Callahan LF, Sloane PD, Devellis RF. Identifying Supports and Barriers to Physical Activity in Patients at Risk for Diabetes. Preventing Chronic Disease. 2006;3(4):A119 16978494 PMC1779283

[40] Penn L, Moffatt SM, White M. Participants' perspective on maintaining behaviour change: a qualitative study within the European Diabetes Prevention Study. BMC public health. 2008;8:235-. doi: 10.1186/1471-2458-8-235 18616797 10.1186/1471-2458-8-235 PMC2491611

[41] King KM, LeBlanc P, Sanguins J, Mather C. Gender-based challenges faced by older Sikh women as immigrants: recognizing and acting on the risk of coronary artery disease. The Canadian journal of nursing research = Revue canadienne de recherche en sciences infirmieres. 2006;38(1):16–40. Epub 2006/05/05. . 16671279

[42] Ferrand C, Perrin C, Nasarre S. Motives for regular physical activity in women and men: a qualitative study in French adults with type 2 diabetes, belonging to a patients' association. Health & social care in the community. 2008;16(5):511–20. Epub 2008/03/22. doi: 10.1111/j.1365-2524.2008.00773.x . 18355245 10.1111/j.1365-2524.2008.00773.x

[43] Sohal T, Sohal P, King-Shier KM, Khan NA. Barriers and Facilitators for Type-2 Diabetes Management in South Asians: A Systematic Review. PloS one. 2015;10(9):e0136202 Epub 2015/09/19. doi: 10.1371/journal.pone.0136202 . 26383535 10.1371/journal.pone.0136202 PMC4575130

[44] Allender S, Cowburn G, Foster C. Understanding participation in sport and physical activity among children and adults: a review of qualitative studies. Health education research. 2006;21(6):826–35. Epub 2006/07/22. doi: 10.1093/her/cyl063 . 16857780 10.1093/her/cyl063

[45] Mier N, Medina AA, Ory MG. Mexican Americans With Type 2 Diabetes: Perspectives on Definitions, Motivators, and Programs of Physical Activity. Prev Chronic Dis. 2007;4(2):A24 17362615 PMC1893123

[46] Paparrigopoulos T, Tzavara C, Theleritis C, Soldatos C, Tountas Y. Physical activity may promote sleep in cardiac patients suffering from insomnia. International journal of cardiology. 2010;143(2):209–11. Epub 2008/12/26. doi: 10.1016/j.ijcard.2008.11.178 . 19108921 10.1016/j.ijcard.2008.11.178

[47] Kredlow MA, Capozzoli MC, Hearon BA, Calkins AW, Otto MW. The effects of physical activity on sleep: a meta-analytic review. Journal of behavioral medicine. 2015;38(3):427–49. Epub 2015/01/19. doi: 10.1007/s10865-015-9617-6 . 25596964 10.1007/s10865-015-9617-6

[48] Hu G, Qiao Q, Silventoinen K, Eriksson JG, Jousilahti P, Lindstrom J, et al Occupational, commuting, and leisure-time physical activity in relation to risk for Type 2 diabetes in middle-aged Finnish men and women. Diabetologia. 2003;46(3):322–9. Epub 2003/04/11. doi: 10.1007/s00125-003-1031-x . 12687329 10.1007/s00125-003-1031-x

[49] Zuo H, Shi Z, Yuan B, Dai Y, Hu G, Wu G, et al Interaction between physical activity and sleep duration in relation to insulin resistance among non-diabetic Chinese adults. BMC public health. 2012;12:247-. doi: 10.1186/1471-2458-12-247 22455464 10.1186/1471-2458-12-247 PMC3342099

[50] Fritschi C, Quinn L. Fatigue in Patients with Diabetes: A Review. Journal of psychosomatic research. 2010;69(1):33–41. doi: 10.1016/j.jpsychores.2010.01.021 . 20630261 10.1016/j.jpsychores.2010.01.021 PMC2905388

[51] Zoladz JA, Pilc A. The effect of physical activity on the brain derived neurotrophic factor: from animal to human studies. Journal of physiology and pharmacology: an official journal of the Polish Physiological Society. 2010;61(5):533–41. Epub 2010/11/18. . 21081796

[52] Chlebowy DO, Hood S, LaJoie AS. Facilitators and barriers to self-management of type 2 diabetes among urban African American adults: focus group findings. The Diabetes educator. 2010;36(6):897–905. Epub 2010/10/27. doi: 10.1177/0145721710385579 . 20974906 10.1177/0145721710385579

[53] Casey D, De Civita M, Dasgupta K. Understanding physical activity facilitators and barriers during and following a supervised exercise programme in Type 2 diabetes: a qualitative study. Diabetic medicine: a journal of the British Diabetic Association. 2010;27(1):79–84. Epub 2010/02/04. doi: 10.1111/j.1464-5491.2009.02873.x . 20121893 10.1111/j.1464-5491.2009.02873.x

[54] Jepson R., Avan G., Bowes A., Harris F., Robertson R., Sheikh A. Physical activity and black and minority ethnic groups: a qualitative study of South Asian people living in Scotland. Scotland: University of Stirling, University of Edinburgh, NHS Health Scotland, 2008.

[55] Al-Kaabi J, Al-Maskari F, Afandi B, Parkar H, Nagelkerke N. Physical Activity and Reported Barriers to Activity Among Type 2 Diabetic Patients in the United Arab Emirates. The Review of Diabetic Studies: RDS. 2009;6(4):271–8. doi: 10.1900/RDS.2009.6.271 . 20043039 10.1900/RDS.2009.6.271 PMC2836198

